# How to Keep Well

**Published:** 1902-02

**Authors:** 


### How to Keep Well.

We have learned that most diseases have their own peculiar circle, their period of invasion, their period of limitation, their crisis and their decline. We have also learned by long experience and repeated trials that these periods may be modified though seldom abridged, and that when fully established usually run their course. By the proper administration of this, or that, as the careful and experienced mariner avoids the rocks and shoals, and outrides the tempest, so the doctor's skill steadies the wavering pulse, allays the fitful fever, soothes racking pain, calms the wild delirium, strengthens the weakened heart, inspires the waning hope, till the eye regains its original lustre, the pulse has lost its irritable thrill, and the silver cord is strengthened that else would have been unloosed. —*Family Doctor.*

